# Phylogenetic characterization of genes encoding for viral envelope glycoprotein (ORF5) and nucleocapsid protein (ORF7) of porcine reproductive & respiratory syndrome virus found in Malaysia in 2013 and 2014.

**DOI:** 10.1186/s12917-016-0933-z

**Published:** 2017-01-05

**Authors:** Seetha Jaganathan King, Peck Toung Ooi, Lai Yee Phang, Zeenathul Nazariah Binti Allaudin, Wei Hoong Loh, Chiou Yan Tee, Shiao Pau How, Lai Siong Yip, Pow Yoon Choo, Ban Keong Lim

**Affiliations:** 1 Department of Clinical Studies, Faculty of Veterinary Medicine, Universiti Putra Malaysia, UPM, Serdang, Selangor 43400 Malaysia; 2 Department of Biotechnology, Faculty of Biotechnology & Molecular Science, Universiti Putra Malaysia, UPM, Serdang, Selangor 43400 Malaysia; 3 Asia-Pacific Special Nutrients Sdn. Bhd, Lot 18B, Jalan 241, Section 51A, Petaling Jaya, Selangor 46100 Malaysia; 4 Vet Food Agro Diagnostic Sdn. Bhd, Lot 18B, Jalan 241, Section 51A, Petaling Jaya, Selangor 46100 Malaysia; 5 Vet Food Agro Diagnostic (M) Sdn. Bhd, Lot 18B, Jalan 241, Section 51A, Petaling Jaya, Selangor 46100 Malaysia

**Keywords:** Porcine reproductive and respiratory syndrome virus, PRRSV, Genetic characterization, ORF5 gene, ORF7 gene

## Abstract

**Background:**

Porcine reproductive and respiratory syndrome (PRRS) is one of the most expensive diseases of modern swine production & results in annual economic losses and cost the industry over 600 million USD in U.S. alone and billions of dollars worldwide. Two atypical PRRS cases were observed in 2013 and 2014 characterized by late-term abortion, fever and sudden increase in sow mortality which persisted for a prolonged period of time.

**Methods:**

Lungs, lymph nodes and other samples were collected for disease investigation. Sequencing of the viral envelope glycoprotein (ORF5) and nucleocapsid protein (ORF7) of PRRSV was done using the BigDye Terminator v3.1 cycle sequencing kit chemistry. The phylogenetic tree was constructed by using the Maximum Likelihood method, generated by Mega 6.06®.

**Results:**

Analysis of the ORF5 and ORF7 showed high degree of sequence homology to PRRSV parent vaccine strain VR-2332, RespPRRSV and other mutant/chimeric virus strains.

**Conclusions:**

Our study suggests that recombination events between vaccine strains and field isolates may contribute to PRRSV virulence in the field.

## Background

Porcine reproductive and respiratory Syndrome (PRRS) is an economically important viral disease that is easily transmitted through direct contact to susceptible pigs and vertically to foetuses. The disease is also known as Mystery Swine Disease, Blue Ear Disease, Porcine Endemic Abortion & Respiratory Syndrome (PEARS) and Swine Infertility Respiratory Syndrome (SIRS) [1, 2]. It is known as one of the most expensive disease of modern swine production. A porcine reproductive and respiratory syndrome virus (PRRSV) outbreak can devastate a herd and determining the origin of the virus can be impossible. PRRS is characterized by an acute viral infection of the porcine macrophage that leads to an immunologically altered state. In extreme cases, respiratory distress, metabolic dysregulation and neuronal involvement result in significant mortality within days to weeks of experimental inoculation with highly pathogenic isolates [3–5]. The virus can also reappear in farms that have taken great lengths to eliminate the virus. Endemic disease from emerging and re-emerging PRRSV results in estimated annual economic losses and the virus is estimated to cost the industry over 600 million USD, or 1.5 million USD per day in the U.S. economy alone [6].

Since its emergence, much has been studied and learned about the virus. It was first detected in North America and reported in 1987 [1]. The virus was then subsequently isolated in Europe in 1990 [7]. Since then it has spread rapidly to Asia and throughout the world. The porcine reproductive & respiratory syndrome virus (PRRSV), the causative agent for the syndrome, is a positive-sense single stranded RNA virus, belonging to the family *Arteriviridae* of the order *Nidovirales*, and genus *Arterivirus* [8, 9].

The PRRSV genome organization is similar to other arterivirus and is approximately 15 kilobases in length. There are 10 open reading frames (ORFs), ORF1a and ORF1b encoding polyproteins that are processed into 14 non-structural proteins (nsp) by viral proteases within the virus genome [10]. The glycosylated membrane associated minor structural proteins GP (2a), GP3 and GP4, respectively are encoded by ORF2a, ORF3 and ORF4 [11]. ORF2b encodes 2b protein, a non-glycosylated structural protein which is virion associated and the principal product of ORF2 [12]. Three major structural proteins, GP5, M and N protein within the virus genome are encoded by ORF5, ORF6 and ORF7, respectively. GP5a, which is referred to as ORF5a protein, is a novel structural protein encoded by an alternative ORF of the subgenomic mRNA encoding GP5 and is incorporated into the virion [13, 14].

Based on genetic characterization, there exist two related but antigenically and genetically distinguishable major genotypes with over 50% RNA sequence variation; the European strain (EU genotype, Type 1, with Lelystad virus as the prototype) representing the viruses predominating in Europe and the North American strain (NA genotype, Type 2, with VR-2332 as the prototype) originally and mostly found in North America [15]. Both genotypes have been described to be evolving independently in Europe and North America and the co-existence of both genotypes has been increasingly evident in several countries, including Malaysia, Thailand, Korea and China [16–20]. Most recently, a variant of genotype 2 also known as highly pathogenic strain of PRRSV, genetically characterized by a unique discontinuous deletion of 30 amino acids (aa) in the non structural protein (Nsp2) of the North American strains was confirmed by the Office International Des Epizooties (OIE) and the Food and Agricultural Organization (FAO) as the causative agent for the severe “high fever” disease designated as the highly pathogenic strain of PRRSV in Asia. Because of its economic significance, a great deal of resource has been invested to research the virus and in developing effective prevention and control strategies. But protocols providing consistent success have been elusive due to the high rate of genetic change and antigenic variability [2, 21–24].

### Situation in South East Asia

**Fig. 1:**
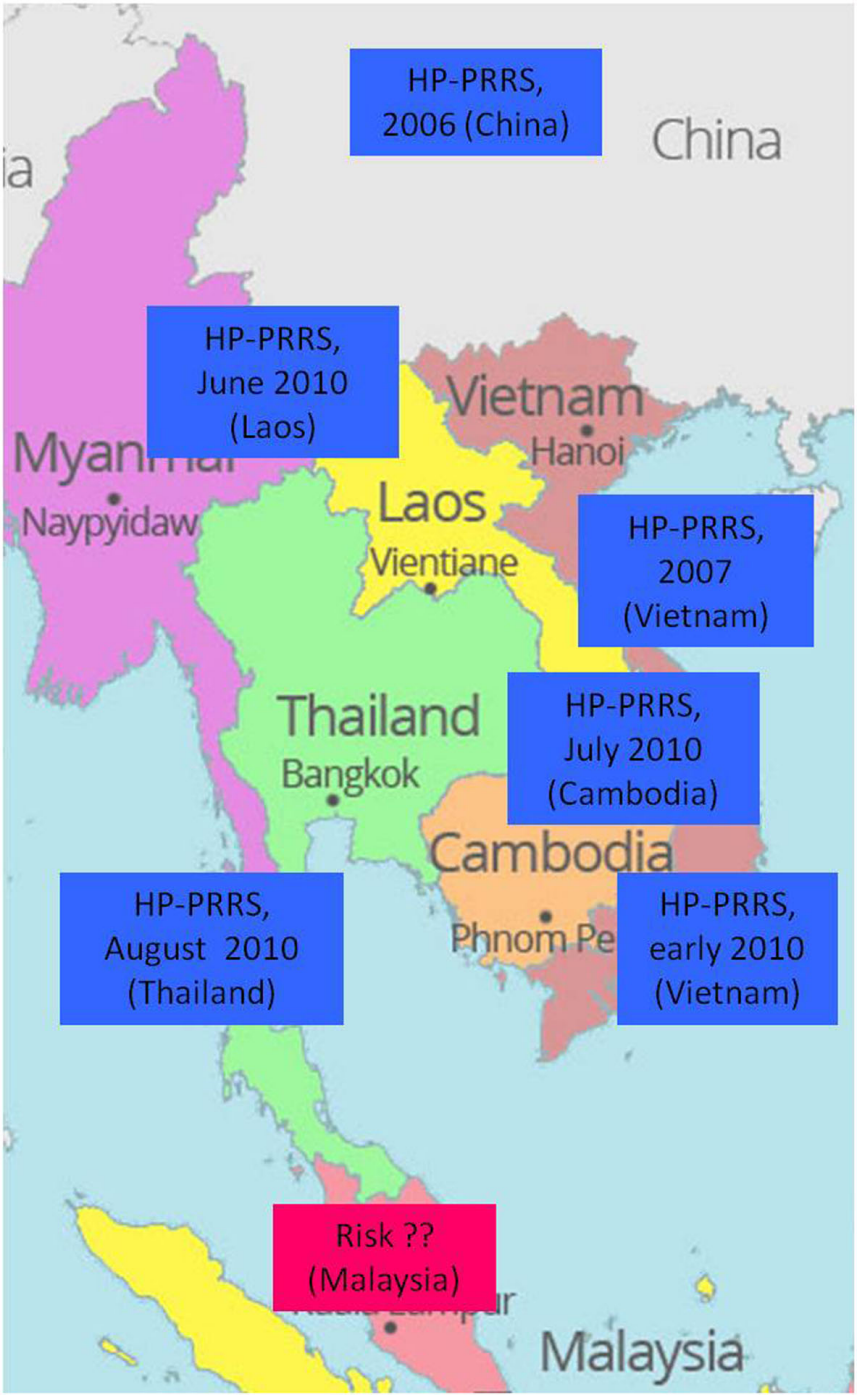
Is Malaysia at risk of HP-PRRSV? Since the disease started in China, it has quickly spread to the surrounding countries in South East Asia. Malaysia is constantly at risk and threat after Thailand reported its first HP-PRRS in 2010

Throughout Asia, PRRS outbreaks were reported in many countries between the late 1980s and early 1990s [9]. The highly pathogenic PRRS (HP-PRRS) which emerged in China in 2006 has spread to South East Asian countries since 2007 [25]. The highly pathogenic PRRS was reported in Vietnam in March 2007 [26], Laos in June 2010 [27], Thailand in 2010 [28], Myanmar in February 2011 [29], Cambodia in August 2010 [30], Philippines in August 2010 [30]. Transboundary spread of HP-PRRSV from southern China to South East Asia suggests that biosecurity failures have occurred, including failure to control animal movements and trading among neighboring countries at borders [31, 32] (Fig. 1).

### Situation in Malaysia

In Malaysia, a syndrome very similar to PRRSV has been recorded in various pig farms as early as 1995 [33]. A serological survey done in about 100 farms in major pig rearing states in the country showed that the pigs found in 93 out of the 100 farms had serological evidence of infection. Subsequently a study done in 2008 showed that 94% of the farms and 83.4% of the pigs were tested positive for PRRSV antibodies [34]. In 2012, another local study documented 89.2% sero-prevalence out of 120 sera collected from 12 non-PRRS vaccinated farms in 6 states. In the same study, 27 tissue samples were collected from 11 farms [35]. Twelve of the tissue samples were positive for PRRSV with all positive for US strains in the selected pig farms. The study concluded that there are more US strains in the selected pig farms, however, it should be noted that there were limitations as the number of samples studied were too small. In 2013, another study conducted in Malaysia concluded that both EU and NA strains are present in Malaysian [36].

### Farming situation in Malaysia

There is approximately 772 farms in Malaysia with 565 farms in West Malaysia with an approximate total no of sows of 0.17 million of which 0.14 million is located in West Malaysia. The estimated total ex farm value of the swine industry in Malaysia is around 2.2 billion ringgit. Similar to the farming situation in other countries, the farming industry in Malaysia has moved on from backyard to industrialized farming systems, with many small farms that has shut down; merged or bought over by bigger players in the industry which explains the reason for the number of farms that have reduced but the farm sizes that are increasing. The farms are mostly open house, farrow to finish and single site, about 10% of the farms use closed house system. The swine respiratory health status varies from state to state and from farm to farm as well. Selangor (located in Central Malaysia) and Penang (located in North Malaysia) are highly urbanized states with limited land space; therefore the farms are close to each other. Johor (located in South Malaysia and borders with Singapore)—these farms used to export to Singapore and are more established. Perak is located between central and north Malaysia which used to be a mining area; the pig farms are located next to lakes and ponds. Sabah and Sarawak which is located in East Malaysia have more land and space, thus, in theory there are less issues.

### Current study

Between 2013–2014, 22 diagnostic cases were received for diagnostic investigation. Seven out of 22 cases received were positive. Six out of the seven cases were positive for NA strain while one case was positive for both EU and NA strain. Among all those cases that were received, there were two atypical cases observed in 2013 and subsequently in 2014 in which high mortality rates were observed in the farm.

#### Case 1: Location: East Malaysia; Year: 2013

##### History & clinical signs

**Fig. 2:**
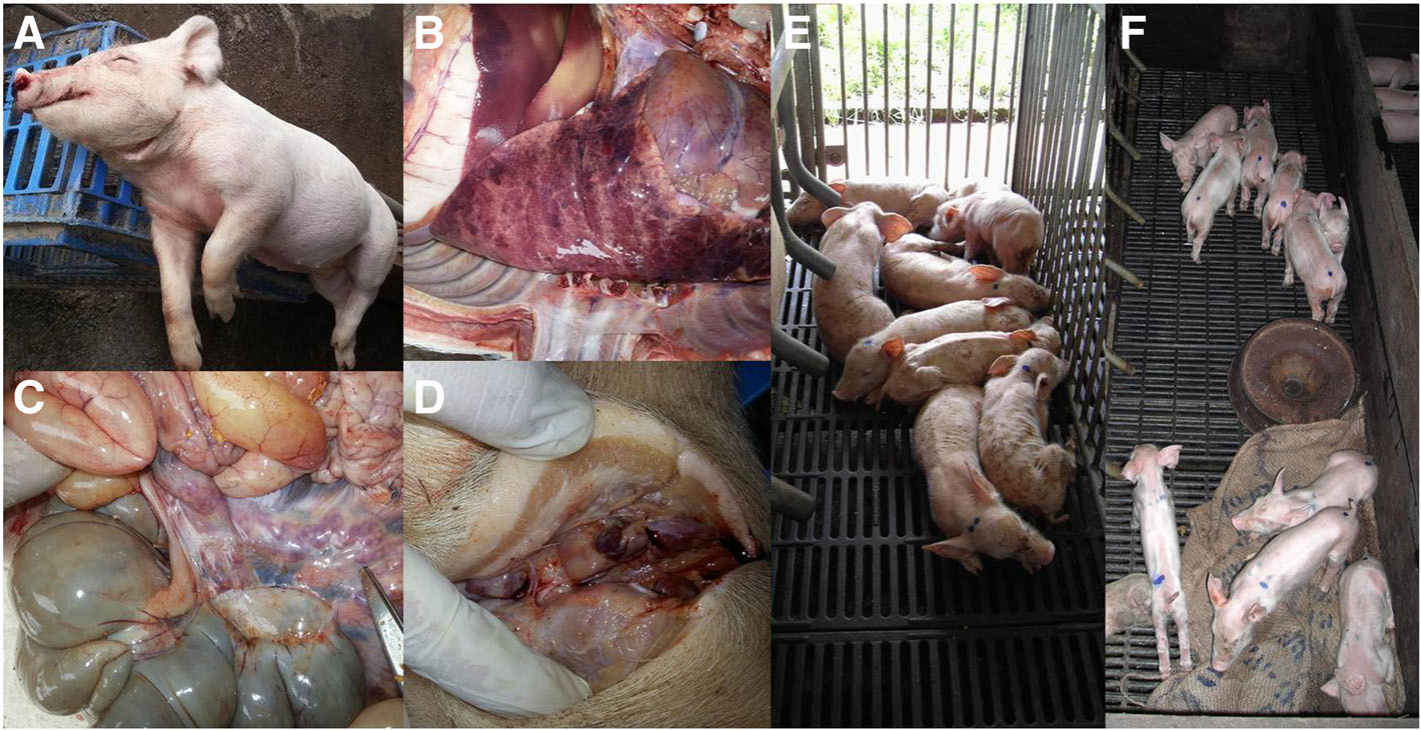
Case 1 (Sar01/2013) East Malaysia. **a**, **b**, **c**, **d**: At the time of sample collection, the case looked like Edema and the animals had secondary bacterial infections. **e**, **f**: The virus had spread to the neighbouring farms and showed similar clinical signs. 3 days old piglets looked weak, runt and emaciated

From November through December of 2012, high mortality was observed in a pig farm in East Malaysia with its return/repeat service increasing by 30%. The piglets were weak and runt. It was also evident that the disease was spreading along to pig farms located in close proximity. About 2–3 months later, there were occurrence of problems in weaners showing signs of Edema and Classical Swine Fever virus and other bacterial infections. At that point, veterinarians were called to assist to collect samples and investigate the disease. Upon tracing back the case history and through differential diagnosis, there were adequate reasons to suspect the case as a potential high fever PRRSV (Fig. 2).

#### Case 2: Location: Central Malaysia, Year: 2014

##### History & Clinical Signs

**Fig. 3:**
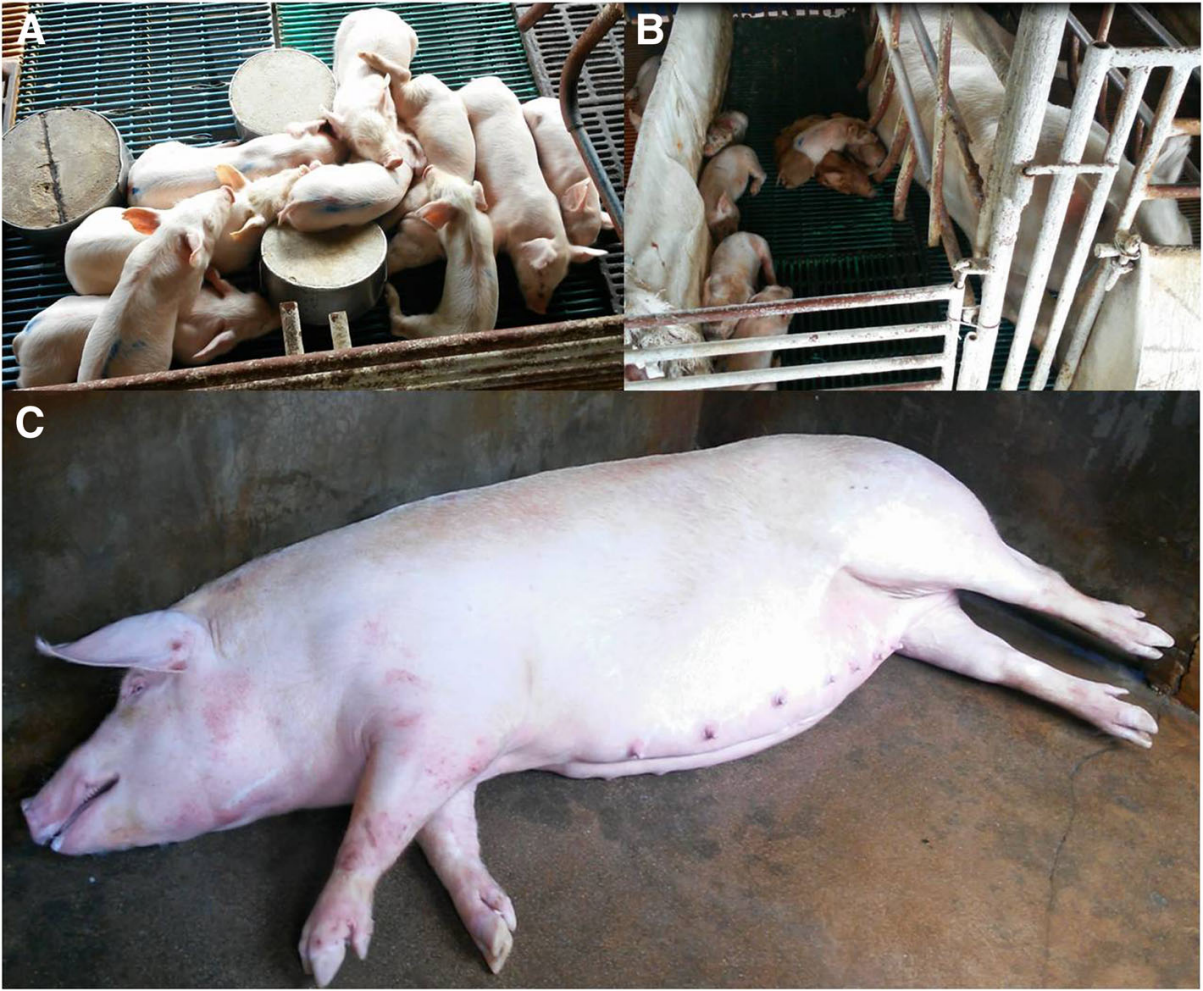
Case 2 (Sel01/2014) Central Malaysia. **a** and **b**: The piglets are weak, runt and chilled. They are clustering together to stay warm. **c**: Sudden death detected in sow with hyperemia and pyrexic

In February 2014, a farrow-finish 300 sow herd reported an outbreak of late-term abortion, 30% of repeat to estrus, and more than 50% of pre-weaning mortality. The sow herd had been regularly vaccinated with Aujeszky’s vaccine, swine fever and PRRS MLV. Mortality of grower & finisher were increased with porcine respiratory disease complex. One week after a schedule blanket vaccination in sow herd with PRRS MLV, there were more than 50% of gilt and first parity sow where sudden death near to term with hyperemia and pyrexia, and 20% of late-term abortion. Umbilical hemorrhage was observed in some cases. Piglet showed ill thrift with periocular oedema and conjunctivitis. Weaners exhibited dyspnea and lethargy and mortality rose to more than 80% (Fig. 3).

**Fig. 4:**
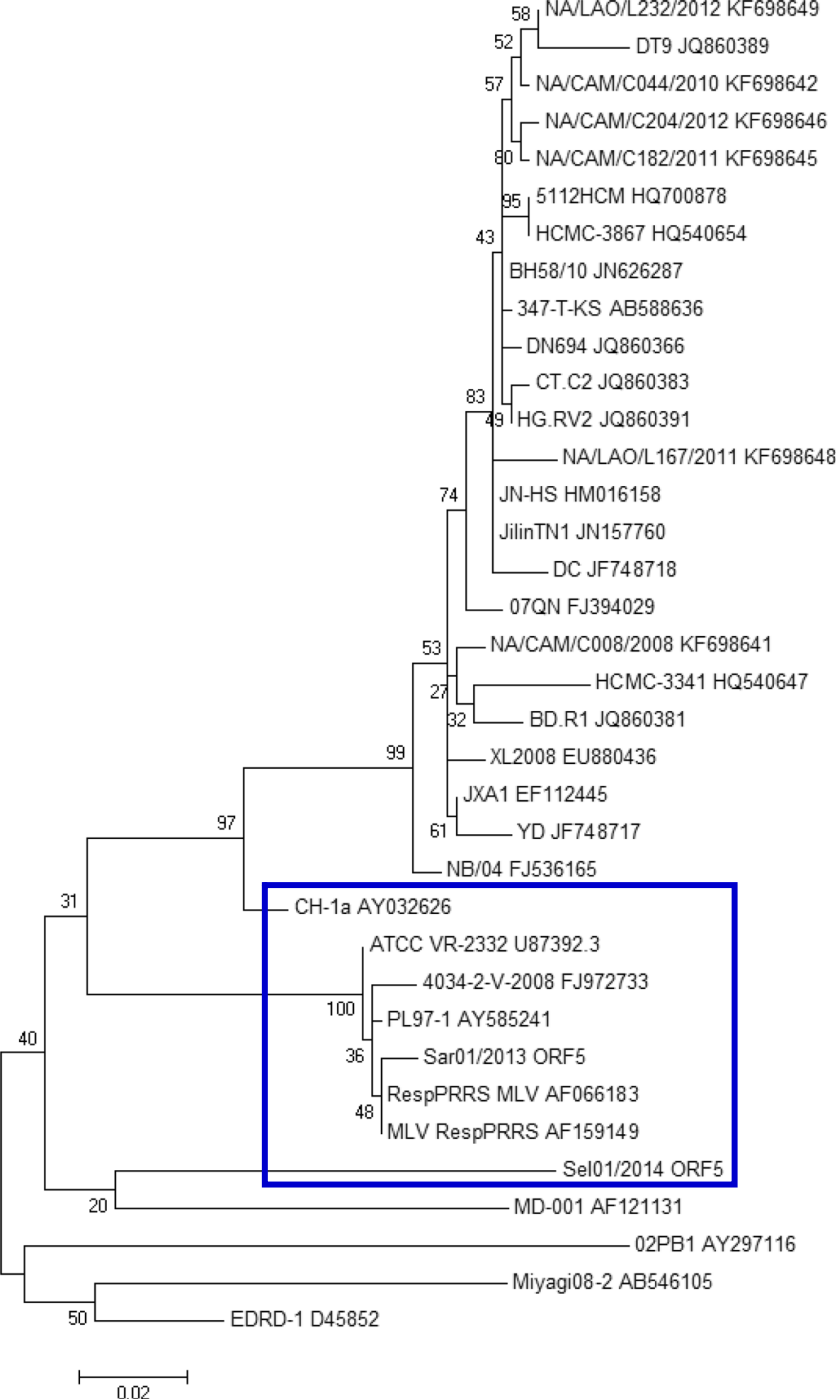
Molecular Phylogenetic analysis of the ORF5 gene of Case 1 (Sar01/2013) & Case 2 (Sel01/2014) from East Malaysia. (Source of Map: http://aseanup.com/free-maps-asean-southeast-asia/)

**Fig. 5:**
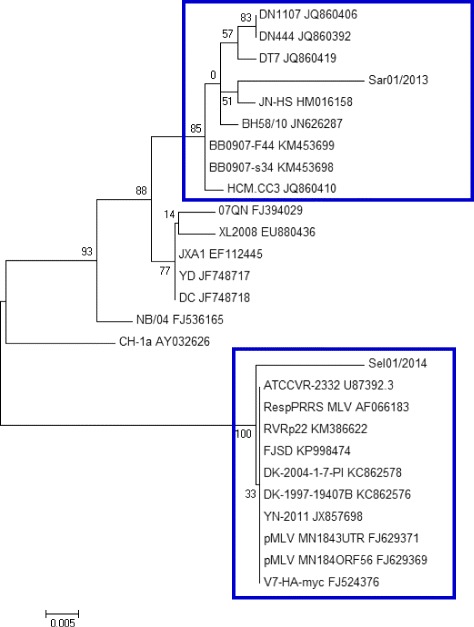
Molecular Phylogenetic analysis of the ORF7 gene of Case 1 (Sar01/2013) & Case 2 (Sel01/2014) from East Malaysia

In this current study, we compare the ORF5 and ORF7 gene sequence from Malaysia with the ORF5 and ORF7 gene isolates from other Asian countries to study the diversity of PRRSV in Malaysia which may help shed some light on the potential origin of PRRSVs in Malaysia. ORF5 encodes the major viral envelope glycoprotein (GP5), which is located on the surface of the virion. GP5 plays an important role in viral infectivity and contains important immunological domains associated with viral neutralization [13, 14]. Several peptide/protein motifs, such as signal peptides, trans-membrane regions, antigenic determinants and glycosylation sites have been widely used for analyzing genetic variation and the molecular epidemiology of PRRSV. The ORF7 encodes the nucleocapsid protein, the most abundant viral protein in virus-infected cells and the most immunodominant antigen in the pig immune response to PRRSV. ORF7 is, therefore a promising candidate for detection and diagnosis [10, 11].

## Results and Discussion

### Analysis of the nsp2 gene of Case 1 and Case 2

After various attempts and optimization strategies, the team did not manage to obtain any positive bands from Case 1 and Case 2 for the nsp2 gene despite getting a positive band from positive controls. It is highly suspected that the nsp2 regions of both cases are highly mutated and not amplifiable with known published primers that are readily available. The nsp2 of PRRSV is a multi-domain protein that has been shown to undergo remarkable genetic variation. From its three major domains [37–39], nsp2 is the most divergent protein between PRRSV types 1 and 2 [40, 41] and also between the pathogenic PRRSV 16244B and an attenuated vaccine and its parental strain VR2332 [40–42]. A large amount of data supports the theory that the middle section of the nsp2 protein is highly susceptible to mutation and tolerant to insertions and deletions, regardless of the pathogenic phenotype of the isolates [39–50]. In fact, reverse genetics experiments have mapped several non-essential viral replication regions in nsp2, including a large deletion of 402 nucleotides in the middle region of the gene [39, 51].

### Analysis of the ORF5 gene (KU512850) of Case 1 (Sar01/2013) from East Malaysia

BLAST analysis of the ORF5 sequence derived from Case 1 (Sar01/2013) from East Malaysia (KU512850) on Genbank showed that the sequence had high similarities to PRRSV sequences from China (KR612142; KR018787), South Korea (KP317086) and USA (KT905092; KT904941). Based on the phylogenetic study, the ORF5 gene sequence derived from Case 1 (Sar01/2013) from East Malaysia (KU512850) clustered together with the ORF5 gene sequences PL97-1 from South Korea (AY585241), 4034-2-v-2008 from South Korea (FJ972733), MD-001 from Taiwan (AF121131) and RespPRRS MLV (AF066183), MLV RespPRRS AF159149 and ATCC VR-2332 U87392.3, the parent strain of the vaccine Ingelvac PRRS MLV. The sequence comparison showed that the nucleotide sequence of the ORF5 gene derived from Case 1 (Sar01/2013) from East Malaysia (KU512850) had 99.3% nucleotide sequence homology with RespPRRS MLV (AF066183) and RespPRRS MLV (AF159149), 99% nucleotide sequence homology with ATCC VR-2332 (U87392.3) the parent strain of the vaccine Ingelvac PRRS MLV and 98% nucleotide sequence homology with 4034-2-V-2008 (FJ972733) from South Korea.

**Fig. 6:**
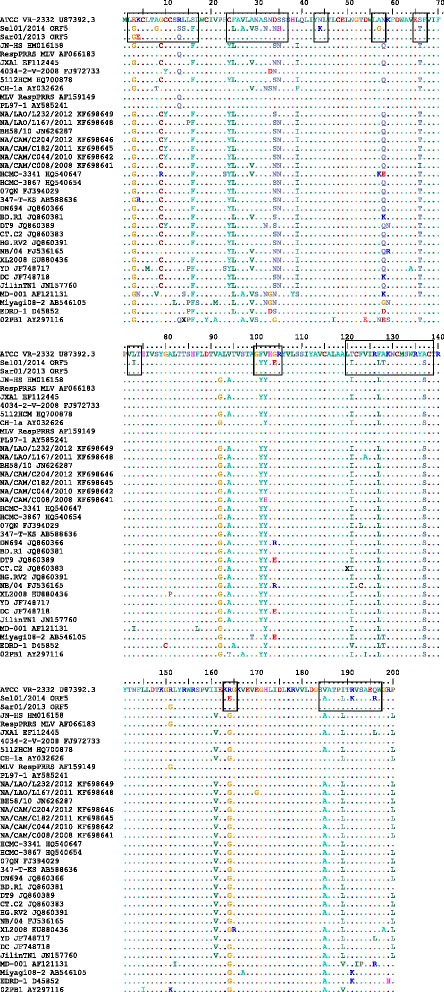
Amino acid sequence alignment of the ORF5 gene of Case 1 (Sar01/2013) and Case 2 (Sel01/2014) from East and Central Malaysia. Identical sequences are *doted. Boxed* portions denote mutations/changes in the amino acid

Three mismatched amino acid changes were observed in position 3, 4 and 13 throughout the ORF5 gene in comparison to ATCC VR-2332 (U87392.3). Amino acid substitutions at positions 3 (Glutamate, E) was substituted with (Glycine, G); amino acid substitution at position 4 (Lysine, K) was substituted with (Glutamate, E) and amino acid substitution at position 13 (Arginine, R) was substituted with (Glutamine, Q) was observed in Case 1 from East Malaysia (KU512850) (Fig. 6).

### Analysis of the ORF7 gene (KU512849) of Case 1 (Sar01/2013) from East Malaysia

BLAST analysis on Genbank shows that the ORF7 gene sequence (KU512849) of Case 1 from East Malaysia derived in this study is highly similar to PRRSV strain JN-HS from Shandong, China, 2008 (Accession No: HM016158). Further analysis by phylogenetic tree studying only selected sequences of the ORF7 gene (KU512849) of Case 1 (Sar01/2013) from East Malaysia with other highly similar sequences from Genbank showed that it clustered with sequences from China (HM016158, KM453699, KM453698), Vietnam (JQ860406, JQ860392, JQ860419, JQ860410) and Laos (JN626287). The phylogenetic tree also suggest that the ORF7 gene (KU512849) of Case 1 (Sar01/2013) from East Malaysia is a derivative of JX-AI from Jiangxi, China (EF112445) which is a representative strain of highly pathogenic PRRSV in China since 2006. The sequence comparison of the nucleotide sequence of ORF7 gene (KU512849) of Case 1 (Sar01/2013) from East Malaysia showed 97.8% sequence homology to to JN-HS from China, 96.7% sequence homology to JX-A1 from Jiangxi, China and 97.5% sequence homology to these sequences from China (HM016158, KM453699, KM453698), Vietnam (JQ860406, JQ860392, JQ860419, JQ860410) and Laos (JN626287). The high genetic similarity between Case 1 (Sar01/2013) and JX-A1 (EF112445) suggest that Case 1 (Sar01/2013) from East Malaysia may be potentially a first HP-PRRSV found in Malaysia. Multiple mismatched amino acid changes were observed in position 15, 46, 91, 109, 117 and 122 throughout the ORF7 gene of Case 1 (Sar01/2013) from East Malaysia in (KU512849) comparison to ATCC VR-2332 (U87392.3).

### Analysis of the ORF5 gene (KU512851) of Case 2 (Sel01/2014) from Central Malaysia

BLAST analysis on Genbank showed that the ORF5 gene sequence derived in this study was highly similar to PRRSV isolate Shizuoka from Japan (Accession No: AB175704.1) and other isolates from America (KT894735.1, U34298.1, DQ477864.1, DQ477718.1). One of the sequence that is highly similar to the ORF5 gene of Case 2 (Sel01/2014) from Central Malaysia is a synthetic contruct clone of PRRSV. Based on the phylogenetic study, the ORF5 gene sequence derived from this study clustered together with the ORF5 gene sequences from Taiwan (AF121131), South Korea (FJ972733; AY585241); RespPRRS MLV (AF066183), MLV RespPRRS AF159149 and ATCC VR-2332 U87392.3, the parent strain of the vaccine Ingelvac PRRS MLV. Sequences for constructing the phylogenetic tree were selected based on genetic relatedness and completeness of sequences that were available on Genbank. The sequence comparison demonstrated that the nucleotide sequence of ORF5 gene derived from Case 2 (Sel01/2014), Central Malaysia (KU512851) has 89.5% sequence homology with sequence MD-001 (AF121131) from Taiwan. The sequence homology of the ORF5 gene from Case 2 (Sel01/2014), Central Malaysia (KU512851) with the vaccine strains were relatively lower than expected with 85.5% with RespPRRS MLV (AF066183), 85.5% with MLV RespPRRS AF159149 and 86% with ATCC VR-2332 U87392.3, the parent strain of the vaccine Ingelvac PRRS MLV. This is not surprising as it has been documented that PRRSV strains differ in virulence [22, 52–55] and vary genetically [56–59] suggesting that the ORF5 gene derived from Case 2 (Sel01/2014), Central Malaysia (KU512851) may be a derivative of the Ingelvac PRRS MLV vaccine, a possible recombinant of the vaccine virus and a wild-type virus, or a truly wild-type virus that is partially homologous to the original parent vaccine strain, VR2332, which may be still circulating in the field [55].

As expected that the ORF5 that encodes for the major envelope glycoprotein would have a high degree of mutation as it is known to be the most variable region [55], multiple mismatched amino acids were observed throughout the ORF5 gene of Case 2 (Sel01/2014), Central Malaysia (KU512851) in comparison to ATCC VR-2332 (U87392.3) (Fig. 4).

### Analysis of the ORF7 gene (KU512848) of Case 2 (Sel01/2014) from Central Malaysia

**Fig. 7:**
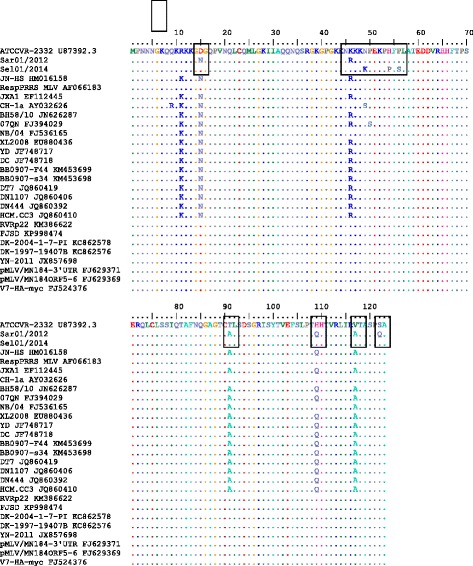
Amino acid sequence alignment of the nucleocapsid protein (ORF7) gene of Case 1 (Sar01/2013) and Case 2 (Sel01/2014) from East and Central Malaysia. Identical sequences are *doted. Boxed* portions denote mutations/changes in the amino acid

BLAST analysis on Genbank of the ORF7 gene sequence from Case 2 (Sel01/2014), Central Malaysia (KU512848) derived in this study demonstrates high similarity to PRRSV strains from South Korea, China, Denmark and USA. Further analysis by phylogenetic tree studying only selected sequences of the ORF7 gene with other highly similar sequences from Genbank suggest that the ancestor for the sequence from this study is ATCCVR2332 which is the parent strain to Ingelvac PRRS MLV vaccine (Fig. 5). The phylogenetic tree also suggest that the ORF7 gene sequence derived from this study groups closely with other sequences from Genbank such as V7-HA-myc (FJ524376), RespPRRS MLV (AF066183), RVRp22 (KM386622), FJSD (KP998474), DK-2004-1-7-PI (KC862578), DK-1997-19407B (KC862576), YN-2011 (JX857698), pMLV/MN184-3UTR (FJ629371) and pMLV/MN184ORF5-6 (FJ629369); two of which are chimeric infectious Porcine reproductive and respiratory syndrome virus type 2 clone representing a background of strain Ingelvac PRRS MLV and 3′UTR of MN184. The sequence comparison confirmed that the nucleotide sequence of ORF7 gene derived from this study is 98.3% similar to ATCC VR-2332 which is the parent strain to Ingelvac PRRS MLV vaccine and has 100% similarity to other sequences as listed in Table 1; two of which are chimeric infectious PRRSV pMLV/MN184-3′UTR FJ629371 & pMLV/MN184-3′UTR FJ629371. Three mismatched amino acid changes were observed in position 49, 54 and 56 throughout the ORF7 gene of Case 2 (Sel01/2014), Central Malaysia (KU512848) in comparison to ATCC VR-2332 (U87392.3) (Fig. 7). At first glance, the four sequences derived from these two cases display a vast diversity in terms of geographical origin of the PRRS virus found in Malaysia. However, when looked closely, all four sequences seem to have one thing in common, they display high sequence homology to the modified live vaccine virus strain regardless of which geographical location the sequences are highly similar to.

**Table 1:** PRRSV isolates derived from this study and other isolates reported previously used for comparison and constructing the phylogenetic tree in this study

No	Isolate Name	Genbank® Accession Number	Gene region	Year	Country	Reference
1	Sar01/2013	KU512850	ORF5	2013	Malaysia	This study
2	Sar01/2013	KU512849	ORF7	2013	Malaysia	This study
3	Sel01/2014	KU512851	ORF5	2014	Malaysia	This study
4	Sel01/2014	KU512848	ORF7	2014	Malaysia	This study
5	NA/LAO/L232/2012	KF698649	ORF5	2012	Laos	Genbank®
6	DT9	JQ860389	ORF5	2012	Vietnam	Genbank®
7	NA/CAM/C044/2010	KF698642	ORF5	2010	Cambodia	Genbank®
8	NA/CAM/C204/2012	KF698646	ORF5	2012	Cambodia	Genbank®
9	NA/CAM/C182/2011	KF698645	ORF5	2011	Cambodia	Genbank®
10	5112HCM	HQ700878	ORF5	2010	Vietnam	Genbank®
11	HCMC-3867	HQ540654	ORF5	2010	Vietnam	Genbank®
12	BH58/10	JN626287	ORF5/ORF7	2010	Laos	Genbank®
13	347-T-KS	AB588636	ORF5	2010	Vietnam	Genbank®
14	DN694	JQ860366	ORF5	2008	Vietnam	Genbank®
15	CT.C2	JQ860383	ORF5	2012	Vietnam	Genbank®
16	HG.RV2	JQ860391	ORF5	2012	Vietnam	Genbank®
17	NA/LAO/L167/2011	KF698648	ORF5	2011	Laos	Genbank®
18	JN-HS	HM016158	ORF5/ORF7	2008	China	Genbank®
19	JilinTN1	JN157760	ORF5	2011	China	Genbank®
20	DC	JF748718	ORF5/ORF7	2010	China	Genbank®
21	07QN	FJ394029	ORF5/ORF7	2007	Vietnam	Genbank®
22	NA/CAM/C008/2008	KF698641	ORF5	2008	Cambodia	Genbank®
23	HCMC-3341	HQ540647	ORF5	2010	Vietnam	Genbank®
24	BD.R1	JQ860381	ORF5	2010	Vietnam	Genbank®
25	XL2008	EU880436	ORF5/ORF7	2008	China	Genbank®
26	JXA1	EF112445	ORF5/ORF7	2006	China	Genbank®
27	YD	JF748717	ORF5/ORF7	2009	China	Genbank®
28	NB/04	FJ536165	ORF5/ORF7	2004	China	Genbank®
29	CH-1a	AY032626	ORF5/ORF7	1996	China	Genbank®
30	ATCC VR-2332	U87392.3	ORF5/ORF7	1995	USA	Genbank®
31	PL97-1	AY585241	ORF5	1997	Korea	Genbank®
32	RespPRRS MLV	AF066183	ORF5/ORF7	2005	USA	Genbank®
33	MLV RespPRRS	AF159149	ORF5	1999	USA	Genbank®
34	4034-2-V-2008	FJ972733	ORF5	2008	Korea	Genbank®
35	MD-001	AF121131	ORF5	1997	Taiwan	Genbank®
36	02 PB1	AY297116	ORF5	2002	Thailand	Genbank®
37	Miyagi08-2	AB546105	ORF5	2008	Japan	Genbank®
38	EDRD-1	D45852	ORF5	1992	Japan	Genbank®
39	DN1107	JQ860406	ORF7	2009	Vietnam	Genbank®
40	DN444	JQ860392	ORF7	2008	Vietnam	Genbank®
41	DT7	JQ860419	ORF7	2012	Vietnam	Genbank®
42	BB0907-F44	KM453699	ORF7	2009	China	Genbank®
43	BB0907-s34	KM453698	ORF7	2014	China	Genbank®
44	HCM.CC3	JQ860410	ORF7	2010	Vietnam	Genbank®
45	V7-HA-myc	FJ524376	ORF7	2010	USA	Genbank®
46	RVRp22	KM386622	ORF7	2014	Korea	Genbank®
47	FJSD	KP998474	ORF7	2015	China	Genbank®
48	DK-2004-1-7-PI	KC862578	ORF7	2004	Denmark	Genbank®
49	DK-1997-19407B	KC862576	ORF7	1997	Denmark	Genbank®
50	YN-2011	JX857698	ORF7	2011	China	Genbank®
51	pMLV/MN184-3UTR	FJ629371	ORF7	2010	USA	Genbank®
52	pMLV/MN184ORF5-6	FJ629369	ORF7	2010	USA	Genbank®

Both cases displayed a high degree of mutation in both genes, ORF5 and ORF7. There are several possibilities that the ORF5 and ORF7 genes from Case 1 (Sar01/2013) and Case 2 (Sel01/2014) are derivatives of the Ingelvac PRRS MLV vaccine, a possible recombinant of the vaccine virus and a wild-type virus, or a truly wild-type virus that is highly homologous to the original parent vaccine strain, VR 2332. This theory was discussed by Opriessnig in 2002 [55].

Further to that, it has been reported that the use of synthetic porcine reproductive and respiratory syndrome virus strain confers unprecedented levels of heterologous protection because current vaccines do not provide sufficient levels of protection against divergent PRRSV strains circulating in the field, mainly due to the substantial variation of the viral genome [60]. However, based on the phylogenetic study and sequence comparison, our study suggest that these synthetic chimeric infectious PRRSV constructs are highly similar to the PRRSV that are causing high mortality rate in the farm in Case 2 (Sel01/2014) in Central Malaysia. Therefore, it is apparent that the rapid rate of PRRSV gene mutation remains a huge challenge for the practicality of such synthetic clone vaccines.

## Conclusion

Over the years, there have been biased and mixed reports of the presence of PRRSV in Malaysia. Based on a collection of studies, it can be confirmed that both European and North American strains are present in Malaysia. However, over the years there have been more North American strains reported compared to European strains. Despite global and intensive use of MLV vaccine, repeated PRRS outbreaks continue due to constant genetic changes in field isolates. Rapid evolution due to high mutation rate has led to new generations of genetically and antigenically variable virus strains in the field. Vaccine strains or derivatives of vaccine strains may also induce disease in the field and persist in vaccinated pigs and spread to non-vaccinated pigs contributing to PRRSV virulence in the field and the inability for effective vaccination. As reported by many other researchers in other countries [55], the results from this study suggest that the virus that persisted in both farms is a product of a recombination event between vaccine strains and field isolates. For future studies, genetic and evolutionary analyses of full length genomes are important to delineate the degree of homology among PRRSVs and for effective vaccine design. We are unable to confirm the presence of highly pathogenic PRRSV in Malaysia. The high degree of nucleotide homology between the local Malaysian isolates with representative strains of highly pathogenic PRRSV strains from China suggest that a derivative of the highly pathogenic virus strain may be present in Malaysia.

## Methods

### Sampling

Two sets of samples from two atypical PRRS cases were sent for disease investigation. One of the sample was from Central Malaysia, Selangor (lungs and lymp nodes) and another from East Malaysia, Sarawak (brains and lungs). The organ samples were pooled for diagnostic testing.

*Animals were humanely slaughtered for disease investigation purpose by professionally trained veterinarians. There was no experimental research done on the animals.*

#### Nucleic acid extraction

Nucleic acid extraction was carried out on the pooled organ samples by using TRIsure® (Bioline®). 100–200 mg of tissue was collected and homogenized using a mortar and pestle and 1 mL of Phosphate Buffered Saline (PBS). 750 uL of cold TRIsure® was added to 250 uL of the homogenized samples in a microcentrifuge tube. The samples were then vortexed vigorously and incubated at room temperature for 5 min. 200 uL of cold chloroform was then added into the same microcentrifuge tube, vortexed vigorously and incubated at room temperature for 5 min. The microcentrifuge tube is then centrifuged at 12, 000 rpm for 15 min at 4 °C. The upper aqueous solution was then transferred to a new and clean microcentrifuge tube. 5 uL polyacryl carrier (Molecular Research Centre Inc) and 500 uL of cold isopropanol are then added and the microcentrifuges containing these reagents were incubated at room temperature for 10 min. After 10 min, centrifuge at 12, 000 rpm for 10 min at 4 °C. Decant the supernatant and wash pellet with 1 mL of 75% cold ethanol. Centrifuge at 12, 000 rpm for 5 min at 4 °C. The pellet was resuspended in 50 uL of TE buffer.

#### PCR amplification of PRRSV—ORF5 and ORF7 gene

The presence of PRRSV in the samples were assessed using a previously described reverse transcriptase nested PCR assay that amplifies a 241 bp nucleotide (European strain) and 337 bp nucleotide (North American strains) respectively [61, 62]. Three sets of primers were used. PLS: 5′-ATG GCC AGC CAG TCA ATC-3′; PLR: 5-TCG CCC TAA TTG AAT AGG TG-3′ [62–64] to reverse transcribes and amplifies a common site in the ORF 7 region of both strains. The nested primer sets for the North American and European Strains were P-US-s: 5′-AGT CCA GAG GCA AGG GAC CG-3′; P-US-as:5′-TCA ATC AGT GCC ATT CAC CAC-3′ and P-EU-s:5′-ATG ATA AAG TCC CAG CGC CAG CGC CAG-3′; P-EU-as:5′-CTG TAT GAG CAA CCG GCA GCA T-3′.

### PCR amplification with ORF5 gene

The primer pairs used were ORF5-F: 5′-ATGTTGGGGAAGTGCTTGACC-3′ and ORF5-R: 5′-CTAGAGACGACCCCATTGTTCCGC-3′ [65].

### PCR amplification with nsp2 gene

The primer pairs used were 2492-F: 5′-GRACTTCCTCARCTTCTTGC-3′ and 3160-R: 5′-TCGACGAGCTTAAAGACCAGA-3′ [51].

### Sequence alignment & phylogenetic analysis of the ORF5 and ORF7 region

Virus sequences were derived from clinical samples sent to the laboratory for diagnostic investigation, with the permission of local veterinary authorities. Virus RNA was extracted using TRIzol reagent according to the manufacturer’s instructions. RNA was quantified by using spectrophotometer (SpectraMax® Plus 384, Molecular Devices). A 603 bp (ORF5) and 337 bp (ORF7) region of the was amplified by reverse transcriptase PCR. The PCR products were purified using the PCR clean-up gel extraction kit according to the manufacturer’s protocol (Macherey-Nagel, Germany). Sequencing of the complete genome of PCV2 was done in a commercial sequencing facility using the BigDye Terminator v3.1 cycle sequencing kit. After sequencing, a Basic Local Alignment Search Tool (BLAST) was performed as a preliminary measure to confirm that all samples were true PRRSV when compared with other sequences deposited in Genbank. The sequence editing and assembly were done by using CLC Workbench. At least one forward and one reverse primer were used to generate a consensus sequence for each gene, which were visually checked for errors prior to alignment using CLC Workbench. Multiple sequence alignments were done by using ClustalW. The phylogenetic tree was constructed by using the Maximum Likelihood method based on the Tamura-Nei model. The percentage of trees in which the associated taxa clustered together is shown next to the branches. Initial tree (s) for the heuristic search were obtained automatically by applying Neighbor-Join and BioNJ algoritms to a matrix of pairwise distances estimated using the Maximum Composite Likelihood (MCL) approach, and then selecting the topology with superior log likelihood value. The tree is drawn to scale, with branches lengths measures in the number of substitutions per site. Positions containing gaps and missing data were eliminated. Evolutionary analyses were conducted in Mega 6 (Biodesign Institute, Tempe, Arizona). The sequence identity matrix data was generated with BioEdit Sequence Alignment Editor version 7.0.5.2 (Tom Hall, US). Sequences used for constructing the phylogenetic tree are listed in Table 1.

### Nucleotide sequence accession numbers

The complete genomic sequences of the ORF5 and ORF7 gene reported in this paper were deposited into the GenBank database under accession numbers KU512848, KU512849, KU512850 and KU512851.

## Abbreviations

ORF: Open reading frame
PCR: Polymerase chain reaction
PRRSV: Porcine reproductive and respiratory syndrome virus
